# Pilot Associations between Adverse Childhood Experiences, Executive Function, and Brain-Derived Neurotrophic Factor (BDNF) among Adults with Excess Adiposity

**DOI:** 10.3390/obesities2030022

**Published:** 2022-08-17

**Authors:** Cindy E. Tsotsoros, Madison E. Stout, Austin R. Medlin, Laurie Wideman, Dolores Vazquez Sanroman, Chibing Tan, T. Kent Teague, Misty A. W. Hawkins

**Affiliations:** 1 Department of Human Development & Family Science, University of Rhode Island, Kingston, RI 02881, USA; 2 Department of Psychology, Oklahoma State University, Stillwater, OK 74074, USA; 3 Department of Health & Wellness Design, School of Public Health, Indiana University, 1025 E. 7th Street, Bloomington, IN 47405, USA; 4 Department of Kinesiology, University of North Carolina Greensboro, Greensboro, NC 27402, USA; 5 OSU Center for Health Sciences, Department of Anatomy & Cell Biology, Tulsa, OK 74107, USA; 6 Integrative Immunology Center, University of Oklahoma, Tulsa, OK 74107, USA; 7 Department of Surgery, University of Oklahoma School of Community Medicine, Tulsa, OK 74107, USA; 8 OSU Center for Health Sciences, Department of Biochemistry and Microbiology, Tulsa, OK 74107, USA

**Keywords:** cognitive function, obesity, overweight, adverse childhood experiences, early life adversity, neurotrophins, neurotrophic factor

## Abstract

Adverse childhood experiences (ACEs) may predict markers of neurocognitive performance (i.e., executive function; EF) and brain health/plasticity (i.e., brain-derived neurotrophic factor; BDNF). This pilot examined: (1) ACES history and current EF performance, (2) ACEs history and current BDNF levels, and (3) current EF performance and BDNF levels. We hypothesized that higher ACEs would be associated with lower EF scores and that these patterns would be associated with serum BDNF levels. Given the pilot nature of the study, emphasis was placed on effect size vs. significance. Participants were 37 middle-aged women. Higher ACEs were not directly associated with EF scores (*β* = 0.08, *p* = 0.635) but showed potentially meaningful negative beta coefficients with proBDNF levels (*β* = −0.22, *p* = 0.200) and positive coefficients with mature BDNF (*β* = 0.28, *p* = 0.094). EF scores and proBDNF showed a positive relationship that did not reach significance (*r* = 0.28, *p* = 0.100) similar to EF scores and mature BDNF (*r* = 0.14, *p* = 0.406). In a modest pilot sample of middle-aged women with excess weight, higher ACEs were potentially associated with lower proBDNF and higher mature BDNF. Larger follow-up studies are warranted given the size of the detected coefficients and theoretical implications of ACEs and obesity as neurocognitively toxic for brain health and performance.

## 1. Introduction

Adverse childhood experiences (ACEs) and obesity are two interrelated and highly prevalent predictors of negative biopsychosocial health outcomes, including relative neurocognitive deficits. ACEs impact an estimated 64% of adults (≥1 ACE) and include traumatic events such as abuse and neglect. Like ACEs, excess adiposity is also widespread among adults, with 72% of adults aged 20 or older qualifying as overweight or obese [1]. Meta-analyses indicate that persons with childhood maltreatment are 1.4 times more likely to develop obesity [2] and that the risk follows a dose-response relationship [3]. ACEs and obesity also show a dose-response effect on health, such that the risk of multiple adverse outcomes increases in a graded fashion as a person’s ACE score and/or obesity levels increases. Such increases may result in neurocognitive injury via various sources, including cognitive deprivation (e.g., in the case of neglect) [4], dysregulation of the body’s stress systems (e.g., hypothalamic-pituitary-adrenal axis alternations), and cardiometabolic and inflammatory biologic changes [5–7]. More recent evidence has linked ACEs and obesity to reduced cortical volume and differences in neural activation of brain regions associated with language, memory, socio-emotional processing, and executive attention and control, as well as neurological comorbidities, including mild cognitive impairment and neurodegenerative disease [4,8–14]. Thus, a growing evidence base identifies neurocognitive function and associated neural health as key factors in the adverse health outcomes associated with ACEs and obesity.

ACEs and obesity are linked to one another, and both appear to impact brain and neurocognitive health independently. Accordingly, indicators of neurocognitive function and brain health (e.g., neuropsychological performance, neurotrophins, white matter integrity, etc.) should be examined in an effort to understand the implications of ACEs history on adult obesity status. One such neurocognitive domain that appears to be highly impactful for obesity onset and progression is executive function (EF), a performance domain of fluid cognition. EF is a subdomain of neurocognition that typically includes the following cognitive abilities: inhibitory control, cognitive flexibility, and working memory. These EF skills are essential for the planning, organizing, problem-solving, initiation, self-monitoring, and inhibition behaviors critical for implementing complex goal-directed behaviors, such as obesity prevention or treatment in an obesogenic environment. Unfortunately, the biologic substrates and candidate mechanisms that characterize high ACEs and fluid cognition or lower EF in obesity are unclear. One possible candidate is neurotrophic factors, such as brain-derived neurotrophic factors (BDNF) [15–18].

BDNF is part of the family of growth factors that also includes the nerve growth factor, neurotrophin-3, and neurotrophin-4/5 [19]. BDNF is synthesized in the cell as a precursor molecule pro-BDNF and later converted into mature BDNF [20]. Indeed, BDNF is the only neurotrophin secreted in an activity-dependent manner which has been shown to be critical for hippocampus-dependent memory in humans [21] and potentially ET. Both BNDF isoforms are biologically active, and some speculation is around their different functional role; therefore, the need to identify which of the BDNF isoforms are related to the ET in the sample of our study [22,23]. BDNF has a long history of being associated with adverse mental health conditions, such as depression. This factor [24] is of most interest to the current study given that its serum/plasma levels, as well as certain BDNF genetic variants (e.g., Val66Met polymorphism rs6265 [25], T-allele carriers for variant rs10767664 [26]), have also been implicated in hippocampal size, memory recovery [21] and the hypothalamic melanocortin pathways that control body weight and response to weight loss interventions and, thus, BDNF likely contribute to functions that control eating, drinking, body weight, and changes to insulin parameters [26–29]. BDNF also changes in response to exercise and/or energy expenditure [30], and lower serum levels have been linked to sedentary behaviors like higher television screen time [31]. In addition to its links with metabolic/energy indicators, BDNF levels may also be useful as indicators of cognitive impairment in young, otherwise healthy adults with obesity [18]. Thus, BDNF may be critical to obesity and its behavioral concomitants via neurocognitive pathways—though it should be noted that null findings for BDNF-obesity relationships have also been reported in the literature [32]. These patterns—if present—may be especially important given previous evidence that higher ACEs have also been linked to lower levels of BDNF in various samples [15–17]. It seems possible that the cognitive and brain alternations observed in individuals with ACEs, obesity, or both may be linked to disruptions in BDNF. However, the relationships between ACEs history and markers of cognitive performance and brain health in individuals with excess weight have not been widely examined. Accordingly, the present study will clarify and advance the science of ACEs and obesity by simultaneously examining the relationships between (1) ACEs history and current EF, (2) ACEs history and current BDNF isoform, and (3) EF and BDNF in a sample with overweight/obesity. This pilot will test if the adverse effects of ACEs are detectable using EF performance and a neurotrophic marker in a preliminary sample of women with excess weight. Such results may point to valuable targets in the shared neurobiological pathways by which ACEs contribute to future obesity. We hypothesized that higher ACEs would be meaningfully associated with lower EF scores. We also hypothesized that higher ACEs and lower EF scores would be meaningfully associated with serum BNDF levels but made no a priori hypotheses about the direction of the associations, given mixed findings in the literature.

## 2. Materials and Methods

### 2.1. Participants

Participants (*n* = 37) were English-speaking women with overweight or obesity (BMI ≥ 25) with no significant medical or psychiatric comorbidities who were seeking weight loss treatment. Exclusion criteria included: (1) not using weight loss medications or participating in a weight loss program, (2) not pregnant or breastfeeding, (3) no history of bariatric surgery, (4) not participated in vigorous exercise in the past 24 h, and (5) not taken any anti-inflammatory medications in the past 24 h. These criteria were included given that these behaviors or conditions may be associated with cognitive function or BDNF levels [33]. See Table 1 for participant characteristics. Basic clinical variables are included in Table 1 as indicators of the health of the sample: fasting glucose, blood pressure, C-reactive protein, interleukin-6, tumor necrosis factor-alpha, and cortisol.

### 2.2. Measures

#### 2.2.1. ACES

Participants completed the ACES checklist, which included 10 different adverse events that may have occurred before the age of 18 [34]. Adverse events include abuse (physical, emotional, and sexual), neglect (physical and emotional), and household dysfunction, which includes having a household member with a substance use or psychological disorder, having a household member incarcerated, having a mother treated violently, and having divorced parents.

#### 2.2.2. Executive Function

Participants completed a portion of the NIH Toolbox-Cognition Battery (NIHTB-CB) [35]. This NIHTB-CB is a neuropsychological battery delivered via iPad that assesses multiple cognitive domains, including executive function, attention, speed, and/or fluid cognition subscales. The NIHTB-CB was created by the NIH and is normed for participants aged 3 to 85 years and was chosen because it is valid, time- and cost-efficient, and allows researchers to compare results across existing NIHTB-CB studies. All tests in the battery have been validated against gold-standard instruments and normed for use in the age range in the current study [35]. The specific tests chosen for the present investigation were Flanker Inhibitory Control and Attention, Dimensional Change Card Sort, List Sorting Working Memory Test, Pattern Comparison Processing Speed Test, and Picture Sequence Memory. This battery takes approximately 25 min to administer. A fluid cognition *T*-score composite (*M* = 50, *SD* = 10) was generated by compiling scores from the aforementioned tests and correcting for age and gender.

#### 2.2.3. Brain-Derived Neurotrophic Factor (BDNF)

Participants provided a fasted blood sample when they arrived in the lab for their baseline assessment. Whole blood was collected via venipuncture into vacutainer tubes to obtain serum per tube manufacturer instructions. Serum was aliquoted and stored in a −80 °C freezer until time of assay. Serum levels of pro- and mature peripheral BDNF (proBDNF and mBDNF, respectively) were analyzed using Biosensis (catalog# BEK2241) [36] human ELISA kits. Cortisol was analyzed using human ELISA kits from R&D Systems. BDNF and cortisol plates were read on a Bio-Rad iMark plate reader. Serum samples were analyzed for levels of CRP, TNFα, and IL-6 using Meso Scale Discovery (MSD) V-PLEX kits and a MESO QuickPlex SQ 120 instrument. Samples were analyzed in duplicate according to the manufacturer’s instructions.

### 2.3. Procedure

Participants were recruited locally through the university and community by online/phone screenings. Eligible participants interested in enrolling provided written informed consent. All procedures were approved by the university’s IRB and adhered to APA ethical guidelines. Demographic variables, ACEs, and eligibility criteria (e.g., medical and psychiatric comorbidities, BMI) were assessed via online surveys. At the start of the baseline assessment, a trained phlebotomist took a fasted blood draw between approximately 8 and 8:30 am. Then, neurocognitive testing was administered by trained research staff. Participants received USD 60 reimbursement for completing the visit.

### 2.4. Data Analysis

First, bivariate associations were run for each key study variable and demographic factors. Then, EF variables (i.e., fluid cognition composite) were regressed on ACES. Next, BDNF variables (i.e., proBDNF and mBDNF) were regressed on ACES. Finally, the BDNF variables were regressed on EF variables. Analyses were run using SPSS (Version 26.0, IBM Corp, 2017) and adjusted for BMI, given that our cognitive composite was already corrected for age and gender.

## 3. Results

Bivariate correlations between all key study variables are presented in Table 2. The variables that were most strongly associated were age and mBDNF, with higher age significantly associated with higher mBDNF level at a strong effect size magnitude (*r* = 0.54, *p* = 0.001). The next strongest associations were the small-to-medium effects observed between both BDNF variables and ACEs (*rs* = −0.22 to 0.26) or the EF fluid cognition composite (*rs* = 0.20 to 0.26). BMI also showed a small-to-medium association with mBDNF (*r* = 0.21).

### 3.1. ACEs and Executive Function

Regressions adjusting for BMI showed that higher levels of ACEs were not directly associated with overall EF fluid cognition composite scores (*β* = 0.08, *p* = 0.635), suggesting that the effect size between adversity in childhood and adult EF scores was not substantial in the present pilot sample with excess adiposity.

### 3.2. ACEs and BDNF

**Figure 1.**
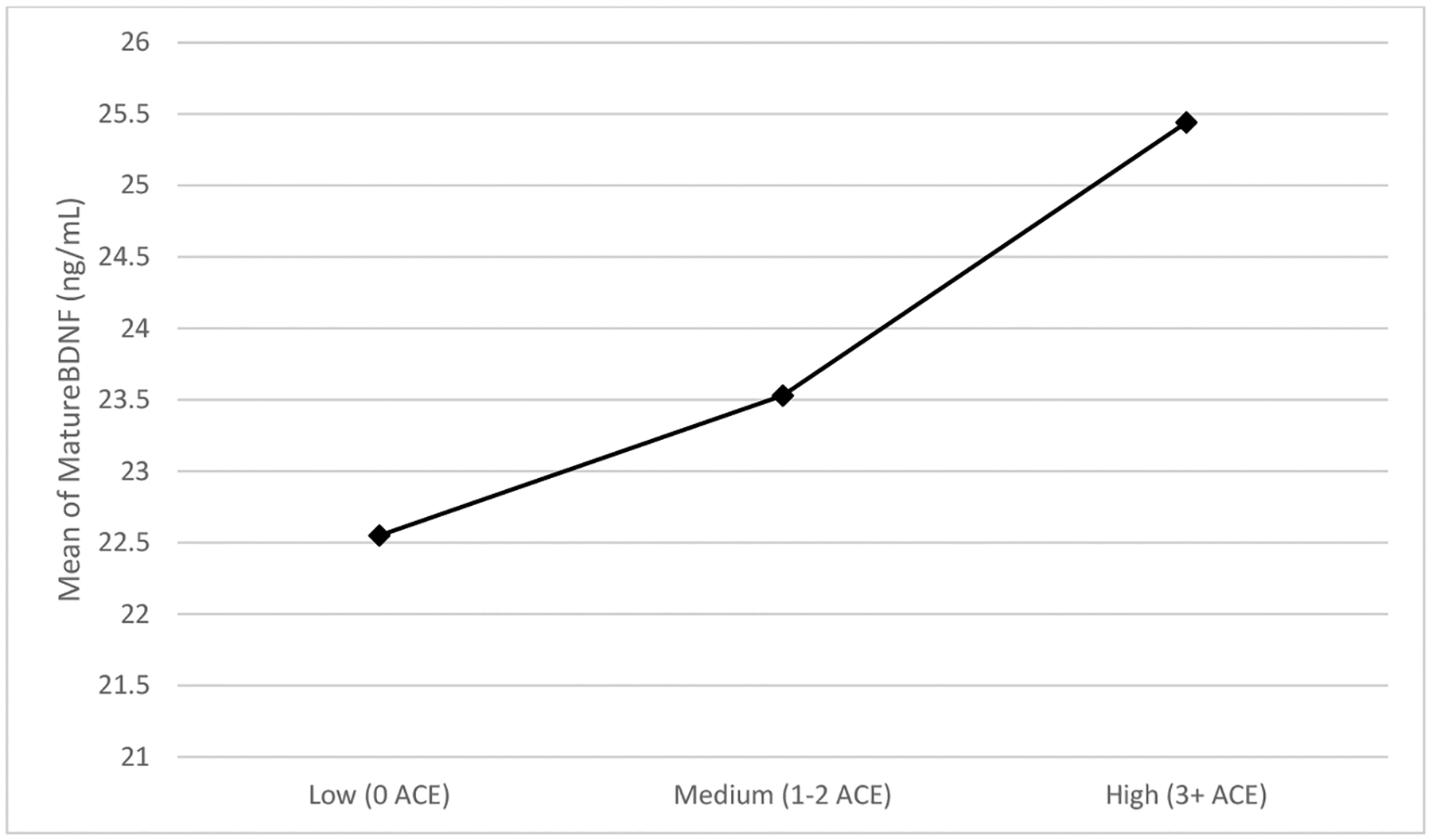

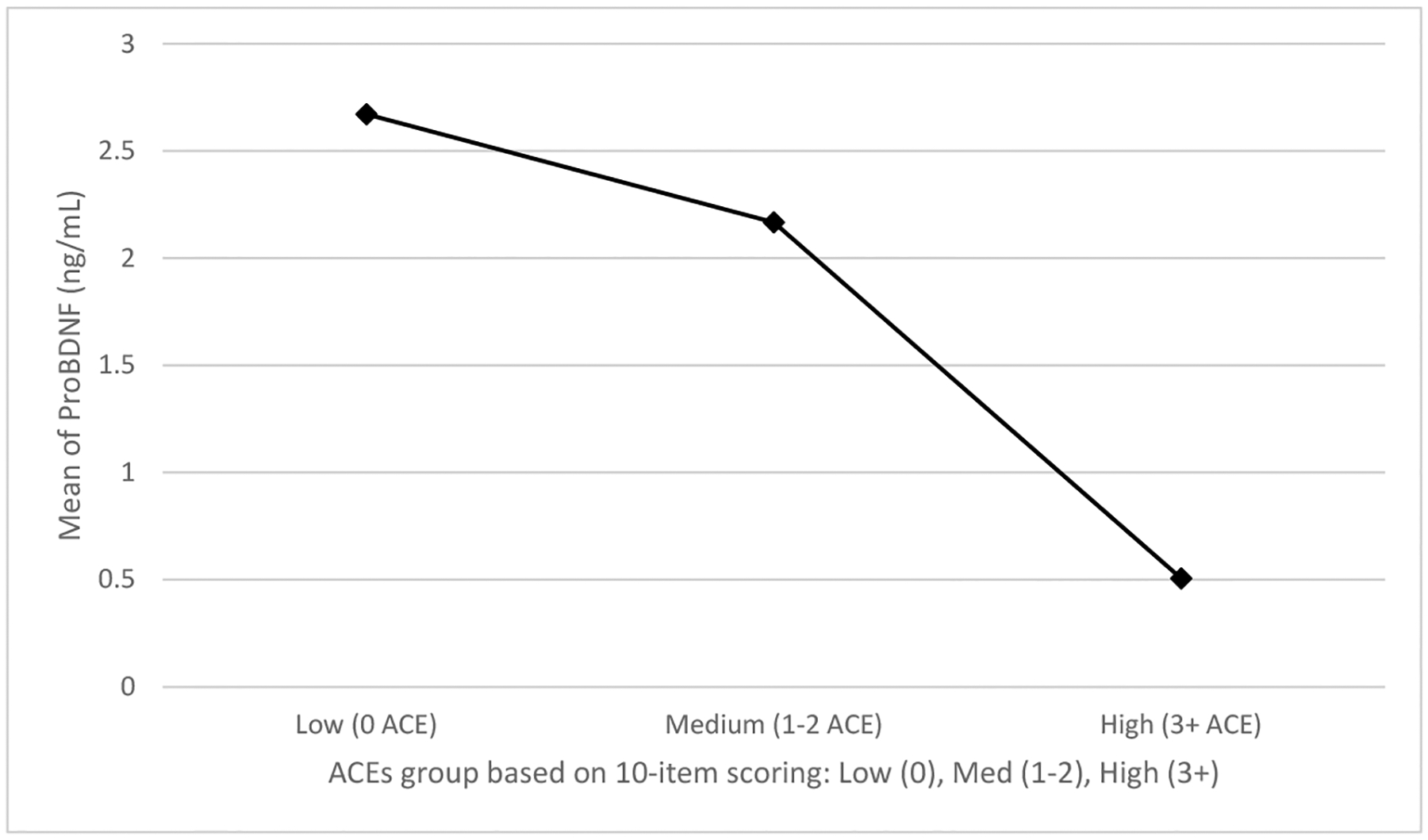
Levels of pro− and mature BDNF across ACEs levels.

ACES did show a meaningful negative effect with non-significant but trending *p*-values for proBDNF levels (*β* = −0.22, *p* = 0.200) and a meaningful positive effect with similar significance levels for mBDNF levels (*β* = 0.28, *p* = 0.094) adjusting for BMI. Figure 1 shows the levels of the pro- and mature BDNF across the different levels of ACE exposure (low = 0, medium = 1–2 ACEs, and high = 3+ ACEs) to show a potential dose-response pattern for these variables. These findings suggest that adversity in childhood may have small-to-moderate and opposing effects on two measures of brain health in adulthood in the present pilot sample over and above any effects of weight status. Such associations might reach significance in a well-powered sample.

### 3.3. Executive Function and BDNF

EF fluid cognition composite scores showed a meaningful positive coefficient that did not reach significance with proBDNF (*β* = 0.28, *p* = 0.100). EF fluid cognition scores showed a smaller positive coefficient that did not reach significance with mBDNF (*β* = 0.14, *p* = 0.406). Again, the magnitude of these effects for proBDNF, especially, could suggest that they may also be detectable and reach significance in a larger sample, suggesting additional study is needed.

## 4. Discussion

The present study advanced the science of ACEs and obesity by simultaneously examining the relationships between ACEs, EF, and BDNF in a pilot sample of women with excess weight and adjusting for weight status. Our first hypothesis that higher ACEs would be associated with poorer EF scores was not supported, as the effect sizes between these variables were not substantive in this sample. This lack of association contrasts with prior literature, showing that higher ACEs are linked to poorer function on EF indices [37,38] and could be due to the restricted range in the EF variable in the present sample and/or the fact that the entire sample exhibited excess adiposity. Specifically, prior work from our laboratory and others has shown that samples with obesity have relatively lower EF performance, especially in the domain of inhibitory control [37,38].

Our hypothesis that higher ACEs would be associated with BNDF levels was supported—in a nuanced pattern—depending on whether examining mature BDNF or its precursor proBDNF. The mature form of BDNF is synthesized from a precursor protein (i.e., proBDNF), which serves different functions than its mature counterpart [20,39,40]. Notably, in this pilot sample, higher ACE levels exhibited negative associations with proBDNF levels but positive associations with mature BDNF, possibly in a dose-response manner for both. This pattern is consistent with previous work in animal models of depression or chronic social defeat. In said animal models, the expression of proBDNF was reduced, but that of mature BDNF was enhanced in brain regions in the reward circuit, such as the nucleus accumbens and amygdala [41]. These seemingly opposing effects make sense in light of the “yin-yang hypothesis” in which BDNF and its precursor cause opposite biological cascades [40].

Specifically, proBDNF is known for its role in synaptic pruning, apoptosis, and promotion of long-term depression (LTD) (i.e., a decrease in synaptic strength), whereas mature BDNF stabilizes neural terminals, signals the receptor tyrosine kinase TrkB, and is essential for early phase long-term potentiation (LTP) (i.e., an increase in synaptic strength) [20]. Thus, our results, if replicated in larger samples, could suggest that individuals with greater adversity in early life might experience higher levels of BDNF as a response to chronic stress. BDNF expression plays a critical role in resilience to chronic stress and is highly dependent on corticosterone secretion [42]. Thus, our study’s “see-saw” tendency of pro- and matureBDNF levels can represent an adaptative response to previous exposure to ACES. Such a pattern in the context of obesity may help elucidate parallel alterations in reward pathways that contribute to an inability to resist palatable foods and/or be hypersensitive to their appetitive properties (e.g., “brake” dysfunction in the context of a sensitive “accelerator”). It should be noted that these BDNF patterns observed in the reward circuit may not be the same as those found for hippocampal or prefrontal cortex brain regions, in which BDNF has been found to be consistently lower [43], and that the present pilot study cannot disentangle whether the pro- and mature BDNF level observed are driven by alternations in specific brain regions, as the study variables were derived from peripheral serum levels of pro- and mature BDNF.

Notably, proBDNF also showed a potentially small, positive relationship with performance on neurocognitive tests of fluid cognition. This finding is consistent with those of Katuri et al. (2021), who also found that lower serum BDNF levels were related to multiple markers of greater cognitive decline in a sample with obesity, including several markers of sustained attention, reaction time, and working memory [18]. Our finding is also interesting in that fluid cognition (and executive function) encapsulates the ability to inhibit so that higher scores can indicate greater inhibitory control. If this finding is replicated and is shown to be significant in a larger sample, it could indicate that the ability to perform well on tasks of inhibition might be paralleled by the brain’s levels of a peptide that has a role in inhibiting synaptic strength.

Although the current brief report and pilot study illuminates some interesting potential pathways linking ACEs with markers of brain health and plasticity in a sample with excess weight, their preliminary and pilot nature requires additional, more extensive studies to clarify the role of BDNF in early life adversity and obesity. Additionally, our study is primarily middle-aged women with excess adiposity, so these patterns may not generalize to younger, older, or other-gendered or weight status populations. Lastly, it would be helpful to prospectively collect data on these topics to clarify the role of weight change on observed associations. Although we controlled for BMI in the present analyses, being able to track weight fluctuations over time and their relations to ACEs history and BDNF expression will be a critical next step.

In conclusion, the current pilot results encourage the following specific recommendations for future work. First, larger follow-up studies in more powered and diverse samples over time are warranted to explore the relationship between ACEs, neurocognitive performance indicators, and neurotrophic levels. Second, the size and opposing directions of the detected coefficients for mature BDNF versus its precursor also call for a nuanced analysis of this biomarker of brain health and plasticity. Failure to examine mature vs. proBDNF levels may help explain inconsistencies in the literature. Third, the potential differential patterns of BDNF response across hippocampal versus reward regions need further explication in the context of weight status. If replicated and extended, the current implications could be that ACEs are neurocognitively toxic for brain health and performance in a manner associated with obesity.

## Figures and Tables

**Table 1.** Participant Demographics, Clinically Relevant Biomarkers, and Key Study Variables (*n* = 37).

	Mean (SD), *n* (%)
*Demographics*	
Age (years)	31.7 (7.933)
Race/ethnicity	26 (70.3%) white
	11 (29.7%) Other/more than one race
	34 (91.9%) Non-Hispanic
Highest level of education	3 (8.1%) Hispanic/Latino/a
	12 (32.4%) Some college/associate’s degree
	16 (43.2%) Bachelor’s degree
	6 (16.2%) Master’s degree
	3 (8.1%) Professional degree
Mother’s highest level of education	3 (8.1%) < High school graduate
	9 (24.3%) High school graduate
	9 (24.3%) Some college/associate’s degree
	9 (24.3%) Bachelor’s degree
	5 (13.5%) Master’s degree
	2 (5.4%) Professional degree
*Clinical Biomarkers*	
Fasting glucose (mg/dL)	97.0 (29.9)
Systolic blood pressure (mmHg)	110.9 (16.2)
Diastolic blood pressure (mmHg)	74.0 (12.7)
C-reactive protein (CRP)	5.2 (5.8)
Interleukin-6 (IL-6)	0.5 (0.4)
Tumor necrosis factor-alpha (TNF-α)	2.4 (1.6)
Cortisol (mcg/L)	10.4 (5.1)
*Key Study Variables*	
BMI (kg/m^2^)	32.54 (6.3)
Body fat (%)	42.78 (5.5)
Total ACE score (1–10)	2.24 (1.98)
Fluid cognition/executive function T-score	49.38 (12.58)
MatureBDNF pre-task (ng/mL)	24.22 (5.08)
ProBDNF pre-task (ng/mL)	1.48 (4.37)

**Table 2.** Bivariate Correlations of Key Study Variables.

	1	2	3	4	5
1. Age	-				
2. BMI	0.17	-			
3. ACES	0.17	−0.09	-		
4. Fluid cognition/executive function^a^	0.15	0.15	0.09	-	
5. proBDNF	0.17	0.07	−0.22^†^	0.26^†^	-
6. Mature BDNF	0.54 **	0.21^†^	0.26^†^	0.20^†^	0.19

*Note*. BMI = body mass index; ACES = Adverse Childhood Experiences Scale;

** *p* < 0.001.

a Fluid cognition/executive function is a composite variable of Flanker Inhibitory Control and Attention, Dimensional Change Card Sort, List Sorting Working Memory Test, Pattern Comparison Processing Speed Test, and Picture Sequence Memory.

† Meaningful effect size ≥ 0.20 (small-to-medium).

## Data Availability

The datasets analyzed for this study can be accessed by contacting the corresponding author.
